# Structural basis of allosteric and synergistic activation of AMPK by furan-2-phosphonic derivative C2 binding

**DOI:** 10.1038/ncomms10912

**Published:** 2016-03-08

**Authors:** Christopher G. Langendorf, Kevin R. W. Ngoei, John W. Scott, Naomi X. Y. Ling, Sam M. A. Issa, Michael A. Gorman, Michael W. Parker, Kei Sakamoto, Jonathan S. Oakhill, Bruce E. Kemp

**Affiliations:** 1 Protein Chemistry & Metabolism, St Vincent's Institute of Medical Research, University of Melbourne, 41 Victoria Parade, Fitzroy, Victoria 3065, Australia; 2 Metabolic Signaling Laboratory, St Vincent's Institute of Medical Research, University of Melbourne, 41 Victoria Parade, Fitzroy, Victoria 3065, Australia; 3 ACRF Rational Drug Discovery Centre, St Vincent's Institute of Medical Research, University of Melbourne, 41 Victoria Parade, Fitzroy, Victoria 3065, Australia; 4 Nestlé Institute of Health Sciences SA, EPFL Innovation Park, bâtiment G, 1015 Lausanne, Switzerland; 5 Mary MacKillop Institute for Health Research Australian Catholic University, Victoria Parade, Fitzroy, Victoria 3065, Australia

## Abstract

The metabolic stress-sensing enzyme AMP-activated protein kinase (AMPK) is responsible for regulating metabolism in response to energy supply and demand. Drugs that activate AMPK may be useful in the treatment of metabolic diseases including type 2 diabetes. We have determined the crystal structure of AMPK in complex with its activator 5-(5-hydroxyl-isoxazol-3-yl)-furan-2-phosphonic acid (C2), revealing two C2-binding sites in the γ-subunit distinct from nucleotide sites. C2 acts synergistically with the drug A769662 to activate AMPK α1-containing complexes independent of upstream kinases. Our results show that dual drug therapies could be effective AMPK-targeting strategies to treat metabolic diseases.

## Abstract (web summary)

AMPK regulates the metabolism and so drugs that activate AMPK might have potential for the treatment of metabolic disease. Here, the authors report the structure of AMPK bound to an activating compound, revealing two binding sites and indicating that dual therapy might be a good drug strategy.

## Graphical abstract

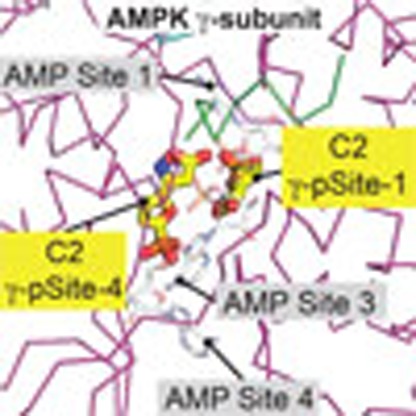

AMP-activated protein kinase (AMPK) is a metabolic stress-sensing kinase responsible for regulating metabolism in response to energy supply and demand. During metabolic stress the cellular AMP/ATP ratio increases leading to activation of AMPK, which in turn switches off energy-consuming anabolic pathways and switches on catabolic pathways to restore ATP levels. The AMPK αβγ heterotrimer comprises a catalytic α-subunit associated with β and γ regulatory subunits (Fig. 1a). Adenine nucleotides (ATP, ADP and AMP) bind interchangeably to the γ subunit where three of the four cystathionine β-synthase (CBS) tandem-repeat sequences provide nucleotide-binding sites^1^. Allosteric activation of AMPK by AMP appears to involve all three sites but site 3 may be the most important^2^,^3^. AMP binding initiates AMPK signalling by promoting phosphorylation of AMPK on α-Thr172 by upstream kinases LKB1 and Ca^2+^/calmodulin-dependent protein kinase 2 (CaMKK2). Once phosphorylated on α-Thr172 AMPK is further activated by AMP, and AMP sustains AMPK signalling by inhibiting dephosphorylation of α-pThr172 (ref. 4). AMP stimulation of αThr172 phosphorylation is dependent on N-terminal myristoylation of the β-subunit, since we demonstrated that AMP stimulation by both upstream kinases is lost following β-G2A mutation^2^. ATP on the other hand opposes allosteric activation by AMP as well as promoting the dephosphorylation of α-Thr172 (ref. 5). The α-regulatory subunit-interacting motif 2 (αRIM2; Fig. 1a)^6^ is required for sensing the adenine nucleotide bound state of the γ-subunit and transducing this signal to the α-catalytic domain, resulting in either stimulation (AMP) or inhibition (ATP) of AMPK activity^1^,^6^,^7^,^8^.

There has been keen interest in developing AMPK-activating drugs for potential therapeutic use in treating metabolic diseases including type 2 diabetes, obesity and cardiovascular disease. A number of small-molecule activators have been identified^9^ and two of these, A769662 (Abbott Laboratories) and 991 (Merck Sharp and Dohme Corporation and Metabasis Therapeutics), were shown in milestone structures to bind to a site formed between the small lobe of the α-subunit kinase domain and the β-subunit carbohydrate-binding module (CBM)^8^,^10^ termed the allosteric drug and metabolite-binding site (ADaM)^11^. Salicylate, the active metabolite of aspirin, is also thought to bind to this site^12^. ADaM site stabilization is enhanced by phosphorylation of the β-subunit residue Ser108 (refs 13, 14). We have shown that the need for β-pSer108 can be bypassed and that dephosphorylated AMPK (or dephosphorylation mimic α1-T172Aβ1-S108Aγ1 mutant) can be activated synergistically by A769662 and AMP whereas there is negligible activity with either activator alone^13^.

Another potent small-molecule allosteric activator of AMPK, 5-(5-hydroxyl-isoxazol-3-yl)-furan-2-phosphonic acid (compound 2, C2, Metabasis Therapeutics), was identified by screening an AMP mimetic library^15^. It is thought that C2 activates both AMPK α1 and α2 isoforms in cell-free assays by binding to the γ-subunit. Although C2 is impermeable to cells, its corresponding di-iso-propyl phosphoester prodrug (compound 13, C13) preferentially stimulates AMPK α1 in mammalian cells^16^. AMP and C2 likely share a common AMPK-activating mechanism as their allosteric effects are not additive, and sensitivity to both is lost in the γ2-R531G AMP-insensitive mutant^16^. The purpose of our study was to identify the C2-binding site(s) on AMPK and investigate the relationship between activation of AMPK by A769662 and C2.

We have determined the crystal structure of the C2: AMPK complex, revealing two C2-binding sites in the γ-subunit distinct from nucleotide sites. C2 acts synergistically with the drug A769662 to activate AMPK α1-containing complexes independent of upstream kinases. Our results raise the possibility that dual drug therapies could be effective AMPK-targeting strategies to treat metabolic diseases.

## Results

### Allosteric activation of AMPK

**Figure 1:**
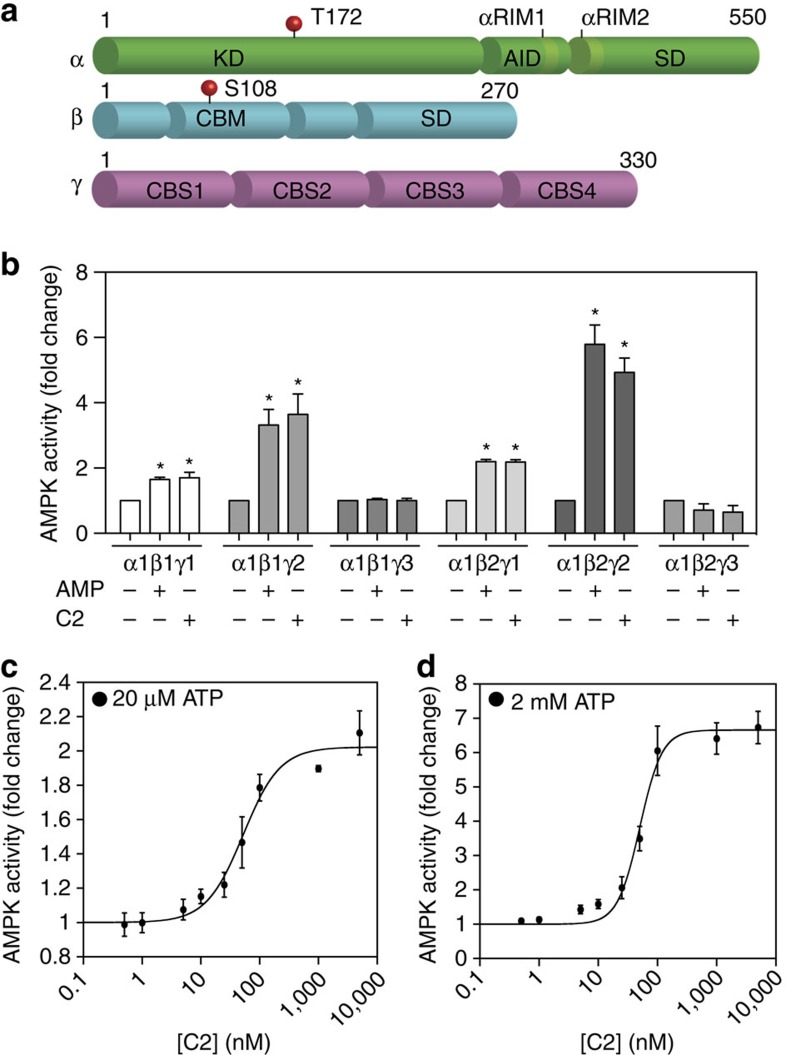
Allosteric activation of AMPK by C2. (**a**) Schematic representation of the three AMPK subunits: the catalytic α-subunit, and the regulatory β and γ-subunits. (**b**) C2 activation of AMPK α1 complexes. Activities of COS7 cell-expressed AMPK (complexes as indicated) were assayed in the absence or presence of C2 (0.1 μM) or AMP (100 μM). Results from triplicate experiments are presented as mean AMPK activity (fold change relative to basal±s.e.m.), with statistical tests conducted using one-way ANOVA. *P<0.05 indicates significant increase in activity compared to untreated control. Effect of ATP concentration on C2 activation of AMPK α1. C2 dose–response (0–5 μM) at (**c**) low (20 μM) ATP and (**d**) near-physiological (2 mM) ATP. Results from six independent experiments are plotted as AMPK activity (fold change±s.e.m.) versus [C2] (nM). The values for EC_50_, fold stimulation and Hill coefficients were calculated based on fitting the data to the equation: Activity=Basal+(((Fold stimulation × Basal)−Basal × [C2]^h^)/ ((EC_50_)^h^+[C2]^h^)). AID, auto-inhibitory domain; CBM, carbohydrate-binding module; CBS, cystathionine β-synthase motif; KD, kinase domain; RIM, regulatory subunit-interacting motif; SD, scaffold domain.

We found that C2 activates both AMPK γ1 and γ2 complexes (α1β1γ1 and α1β2γ1; α1β1γ2 and α1β2γ2) to a similar degree as AMP, whereas neither C2 nor AMP-activated γ3-containing complexes (α1β1γ3 and α1β2γ3) (Fig. 1b). Lack of AMP activation of γ3-containing complexes has also been reported recently^17^ but not in all studies^18^,^19^.

Since ATP concentration was previously shown to alter AMP dependence^5^, we investigated C2 activation of AMPK α1 at low (20 μM) and physiological (2 mM) ATP concentrations. Comparison of dose–response curves revealed that the half-maximal concentrations for C2 activation were similar at both low and high ATP (52.3±7.9 and 50.3±4.4 nM for 20 μM and 2 mM ATP, respectively; Fig. 1c,d), implying that C2 and ATP binding is non-competitive. In contrast, previous studies have shown that the concentration of AMP required for half-maximal activation increases with higher concentrations of ATP, which is consistent with AMP/ATP competition at the γ-subunit allosteric sites^5^. We also observed that C2 activation becomes strongly co-operative at high ATP, as shown by a shift in the Hill coefficient from 1.4±0.3 (20 μM ATP) to 2.3±0.4 (2 mM ATP) (Fig. 1c,d). In contrast, AMP does not exhibit co-operative binding at high ATP concentrations (Hill coefficient 1.0±0.2; Supplementary Fig. 1 and Supplementary Table 1). We are uncertain of the structural basis for the effect of ATP on C2 co-operative binding as well as the kinetic basis for ATP not influencing AMP activation in a co-operative manner. The γ-subunit resembles a classic multimeric-enzyme with its 4 CBS tandem repeats. One interpretation is that ATP binding to the γ-subunit influences C2 co-operativity but we have been unable to obtain crystals with both C2 and ATP bound to the γ-subunit thus far. In the event ATP cannot bind to γ in the presence of C2 we cannot formally eliminate the possibility that ATP binding at the kinase active site (α-subunit) rather than the γ-subunit may induce C2 co-operative binding.

### Synergistic activation of AMPK

**Figure 2:**
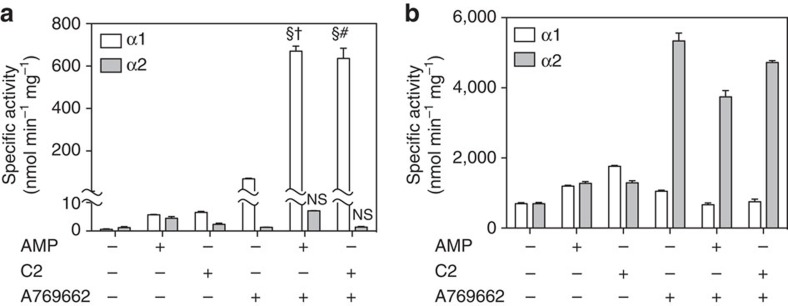
C2/A769662 synergistic activation of AMPK. Wild-type AMPK α1 and α2 were pre-treated with (**a**) λ-phosphatase or (**b**) CaMKK2, and assayed in the absence/presence of AMP (100 μM) and C2 (0.1 μM), either individually or in combination with A769662 (20 μM). Results from triplicate experiments are presented as mean specific activity (nmol min^−1^ mg^−1^±s.e.m.), with statistical tests conducted using one-way ANOVA Sidak's multiple comparisons test. Symbols §, † and #; *P*<0.01 indicate significant increase in activity compared with A769662, C2 and AMP controls, respectively, while NS indicates that there was no significant difference between the co-incubated samples and their individual control samples.

We examined whether C2, like AMP, can synergize with A769662 to activate dephosphorylated AMPK (Supplementary Fig. 2a). Furthermore, we explored whether this synergistic activation is also present in the AMPK α2 isoform. Modest allosteric activations were observed with λ-phosphatase pre-treated AMPK α1 or α2 complexes individually incubated with AMP or C2 or A769662 (Fig. 2a). We attribute the larger A769662 response using AMPK α1 to residual β1-Ser108 phosphorylation persisting after λ-phosphatase treatment (Supplementary Fig. 2a). Synergistic activation occurred when the λ-phosphatase-treated AMPK α1 (basal activity ≈0.6 nmol min^−1^ mg^−1^) was co-incubated with combinations of AMP/A769662 (≈671 nmol min^−1^ mg^−1^ or≈1100-fold) and C2/A769662 (≈636 nmol min^−1^ mg^−1^ or ≈1040-fold), but not with CaMKK2-treated AMPK α1 (Fig. 2a,b; Table 1). It should be noted the maximum activity observed in C2-stimulated, CaMKK2-treated AMPK α1 is ≈threefold higher than the maximum activity observed with the λ-phosphatase-treated AMPK α1 co-incubated with C2/A769662. Moreover, the synergistic activation was specific to AMPK α1, as the λ-phosphatase-treated AMPK α2 (basal activity ≈1.2 nmol min^−1^ mg^−1^) co-incubated with AMP/A769662 or C2/A769662 was relatively insensitive to dual ligand activation (AMP/A769662 activation ≈7.2 nmol min^−1^ mg^−1^ or ≈6.1-fold; C2/A769662 activation ≈1.3 nmol min^−1^ mg^−1^ or ≈1.2-fold; Fig. 2a; Table 1). Similarly to CaMKK2-treated AMPK α1, the CaMKK2-treated AMPK α2 did not exhibit dual synergistic activation by both AMP/A769662 and C2/A769662 (Fig. 2b).

### Novel C2-binding site in the γ-subunit

**Figure 3:**
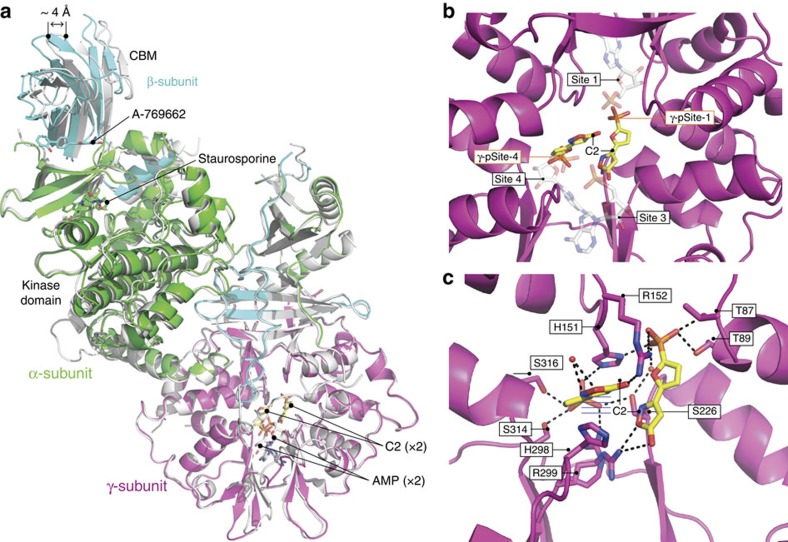
C2 binds the γ-subunit of AMPK. (**a**) Cartoon representation of the AMPK heterotrimer (α2β1γ1) bound to C2 (yellow sticks) in the γ-subunit (magenta), Staurosporine bound to the α-subunit (green) and A769662 bound at the ADaM binding site at the interface of the α-kinase domain and the CBM of the β-subunit (cyan) superimposed with the AMP-bound heterotrimer (white). Arrow shows the approximate distance between equivalent βCBM residues in the C2-bound and AMP-bound heterotrimers. (**b**) The position of C2 with respect to CBS sites, with AMP superimposed (from PDB 4CFE). (**c**) AMPK interactions (black dashed lines and blue parallel lines for π stacking) with C2 (yellow sticks). Key residues (labelled) are modelled as sticks. Note that γ-Arg299 was modeled in two discrete conformations.

To gain further insight into the mechanism of C2-mediated AMPK activation we solved the X-ray crystal structure of full-length α2β1γ1 isoform co-crystallized with C2 and AMP to 2.99 Å resolution (Table 2). The structure revealed two molecules of C2, bound within the solvent-accessible core of the γ-subunit. The phosphate groups of both C2 molecules overlap the phosphate binding sites of AMP in sites 1 and 4, while the 5-(5-hydroxyl-isoxazol-3-yl)-furan moieties occupy novel binding sites independent from the AMP-binding sites (Fig. 3a–c; Supplementary Fig. 3a). The asymmetric unit contained two AMPK heterotrimers. One heterotrimer contained AMP bound at each of CBS sites 3 and 4 (Fig. 3a, white) whereas the other heterotrimer was devoid of AMP and fortuitously contained two molecules of C2 instead (Fig. 3a–c). The two distinct liganded complexes allowed us to comprehensively probe the differences between AMP and C2 binding to AMPK.

Our AMP-bound heterotrimer closely resembles the previously published AMP-bound α2β1γ1 structure (PDB 4CFE)^8^. The two molecules superpose with a r.m.s. deviation of 0.55 Å over 884 Cα atoms, with key AMP-interacting residues having the same conformation. In contrast, the C2-bound molecule has an r.m.s. deviation of 0.75 Å (over 864 Cα atoms) when superimposed with 4CFE^8^. The largest structural difference between the C2 and AMP-bound heterotrimer is in the β-CBM and the N-lobe of the kinase domain, where a shift of up to 4.0 Å occurs (Fig. 3a). This shift reflects the formation of a canonical kinase salt bridge, formed between conserved residues α-Lys45 and α-Glu64, in the C2-bound heterotrimer. This important interaction is regarded as an active kinase conformation signature^20^ and is conspicuously absent in previous α2β1γ1 structures^8^. The C2-bound γ1-subunit conformation closely mimics that of the AMP-bound complex. Despite the considerable chemical differences and different binding orientations between C2 and AMP the side chains of the γ1-subunit residues are remarkably similar in conformation, with the notable exceptions being γ-Arg70 and γ-His298 (Supplementary Fig. 4a,b). A region that does distinguish between the two heterotrimers is the nucleotide sensing α-subunit RIM2 motif^1^,^6^,^8^. Strong electron density was observed for the α-RIM2 in the AMP-bound heterotrimer, but only very weak electron density was observed for α-RIM2 in the corresponding C2-bound heterotrimer and could therefore not be included in this model (Supplementary Fig. 3b). This is a C2 specific effect as the previously published AMPK structure 4CFE (crystallized in the same space group (*P*21) and solved to an equivalent resolution of 3.02 Å) revealed strong electron density for both AMP-bound heterotrimers in the asymmetric unit^8^. Importantly, these data are consistent with the α2-subunit RIM2 being incompletely engaged with the γ-subunit when C2 is bound.

The two C2 molecules occupy the interface between the CBS-binding sites 1, 3 and 4, with the phosphate group of C2 occupying a similar position to that of AMP-bound phosphate group in sites 1 and 4 (Fig. 3b). For this reason we have named specific C2-binding sites γ-pSite-1 and γ-pSite-4, referring to the equivalent AMP phosphate group position (Fig. 3b,c).

In both sites the C2 molecule makes six protein-mediated hydrogen bond contacts, while C2 in γ-pSite-4 makes an additional π-stacking interaction with γ-His298 (Fig. 3c). All of the residues that contact C2 are shared with AMP, yet the binding constant for C2 is >100-fold higher than that for AMP^15^,^16^. The basis of C2's increased affinity is not obvious to us.

### Mutational analysis of the C2-binding site

**Figure 4:**
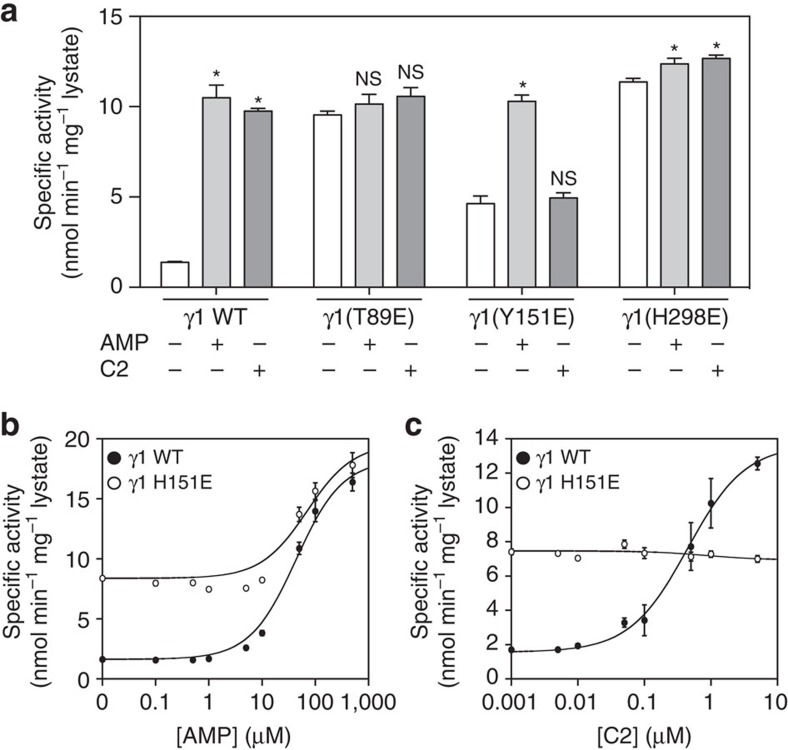
Effect of AMPK γ-subunit mutations on C2 activity. AMPK α1β1γ1 phospho-mimetic T172D and their respective γ mutants T89E, H151E, and H298E were expressed as FLAG-α fusion proteins in COS7 mammalian cells. (**a**) Activities of AMPK mutants were tested in the absence or presence of C2 (5 μM) or AMP (100 μM). Results from four independent experiments are presented as specific activity (nmol min^−1^ mg^−1^ lysate±s.e.m.), with statistical tests conducted using one-way ANOVA. *P<0.05 indicates significant increase in activity compared with untreated control. (**b**) AMP dose–response of AMPK α1-T172D γ1 wild-type and γ1-H151E mutants (0–500 μM). The data was plotted as AMPK activity (nmol min^−1^ mg^−1^ lysate±s.e.m.) versus [AMP] (μM) from four independent experiments. (**c**) C2 dose–response of AMPK α1-T172D γ1 wild-type and γ1-H151E mutants (0–5 μM). Curve was plotted as AMPK activity (nmol min^−1^ mg^−1^ lysate±s.e.m.) versus [C2] (μM) from four independent experiments. The values for EC_50_ and fold stimulation were calculated based on fitting the data to the equation: Activity=Basal+(((Fold stimulation × Basal)−Basal × [C2])/((EC_50_)+[C2])).

To validate the C2-binding sites, we expressed three AMPK γ1 mutants (T89E, H151E and H298E) in COS7 mammalian cells and measured their sensitivity to C2 or AMP. We initially found that all the γ1 mutants displayed increased but variable Thr172 phosphorylation and basal activities compared with the wild-type (WT) γ1-containing AMPK complexes. We therefore co-expressed the mutants with the phospho-mimetic AMPK α1(T172D), which is more amenable to studying the effect of these γ mutations on allosteric activation (Supplementary Fig. 2b)^5^,^21^. C2 and AMP activated the AMPK α1(T172D) β1γ1 enzyme to a similar extent (≈ 8- and 10-fold, respectively; Fig. 4a). The γ-T89E and γ-H298E mutants had increased basal activities (9.5±0.2 and 11.4±0.2 nmol min^−1^ mg^−1^ lysate for γ-T89E and γ-H298E, respectively) in the absence of C2 and AMP compared with γ1 WT (1.4±0.1 nmol min^−1^ mg^−1^ lysate), and were insensitive to further activation by either C2 or AMP (Fig. 4a). It is possible that substituting for a negatively charged glutamate residue at the T89 and H298 positions mimics the effect of the phosphate groups of AMP or C2, resulting in constitutive activation. This would also explain the increased Thr172 phosphorylation observed with these mutants. The γH151E mutation completely abolished AMPK activation by C2 (Fig. 4a,c), while only partially reducing AMP-dependence and sensitivity compared to the wild-type control (Fig. 4a,b).

### C2-induced conformational rearrangements of the γ-subunit

A superposition of the two AMPK heterotrimers reveals two major side-chain conformational changes between AMP and C2 binding. Firstly, the imidazole ring of the aforementioned γ-His298 has flipped 180° and rotated at the Cβ>90° (∼ 6.0 Å) to form a π-stacking interaction with C2 in γ-pSite-4 while simultaneously blocking site 3, mimicking ATP-bound AMPK (PDB 4EAK)^3^ (Supplementary Fig. 4a). The conformational difference of the γ-His298 side chain is critical for AMP or C2 binding. Secondly, the position of C2 at γ-pSite-1 causes the side-chain guanidine group of γ-Arg70 to shift approximately 4 Å away from the AMP-bound conformation. In this new conformation, γ-Arg70 would clash with critical AMP-sensing residue α-Glu368 from the αRIM2 (Supplementary Fig. 4b). The clash may cause α-Glu368 to lose hydrogen bonds to site 3 AMP-interacting residues γ-Arg70 and γ-Lys170, that tether α-Glu368 to the γ-subunit^1^,^6^,^8^. In turn, this causes the αRIM2 disengagement. The loss of RIM2 association with the γ-subunit leads to the loss of protection from α-Thr172 dephosphorylation and a concomitant reduction in Vmax with C2 as shown by Hunter *et al.*^16^. In support of this proposal, previous studies have shown that α-Glu368 mutation results in a comparable reduction in Vmax with AMP stimulation and α-Thr172 dephosphorylation protection^6^,^7^,^8^.

### AMPK isoform specificity of C2 allosteric activation

**Figure 5:**
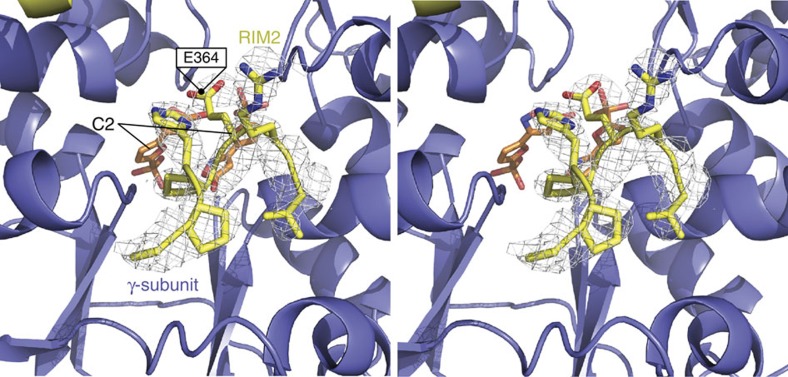
Omit map of the α1RIM2 with C2 bound in the γ-subunit. Stereo 2*F*_*o*_*–F*_*c*_ omit map of the α1RIM2 (yellow) from the heterotrimeric human AMPK RIM swap chimaera (α2 1-347/α1 349-401/α2 397-end β1γ1) bound to C2, contoured at 1σ. Continuous density (white mesh) can be seen for seven residues of the α1RIM2, including E364 (α2 E368). Weak density for the side-chain carboxyl of E364 suggests some interaction with C2 (orange sticks) bound γ-subunit (blue).

Secondary structure elements of αRIM2 are crucial for the isoform specificity of C2. Structural comparison of α1 and α2 isoform αRIM2/γ-subunit interactions reveal the α-helical α1RIM2 (PDB 4CFH)^1^ forms a strong interaction (three hydrogen bonds and six salt-bridges)^22^ with the γ-subunit in a groove between γ-helix α10 and γ-helix α4, formed upon AMP or C2 binding (Supplementary Fig. 3b,c). In contrast, α2 isoforms have a loop motif (PDB 4ZHX and 4CFE)^8^ that forms a single hydrogen bond and four salt-bridges^22^ with the γ-subunit (Supplementary Fig. 3b,c). We propose the weaker interacting α2 isoform αRIM2 is incompletely engaged upon C2 binding relative to the α1 isoform, resulting in the α-isoform specificity for allosteric and synergistic activation and protection from α-Thr172 dephosphorylation. We solved the X-ray crystal structure of a heterotrimeric α2/α1 RIM chimaera, α2(1-347)/α1(349-401)/α2(397-end) β1γ1 (which has previously been shown to completely restore maximal allosteric activation and C2-mediated protection of α-Thr172 dephosphorylation^16^), co-crystallized with C2 to 2.99 Å (pdb: 5EZV; Table 2). Unlike the aforementioned crystal structure (4ZHX), α2/α1 RIM swap chimaera was crystallized in the absence of AMP, revealing two C2-bound AMPK heterotrimers in the asymmetric unit. Both of the C2-bound heterotrimers (5EZV) closely resembled the previous C2-bound heterotrimer (molecule 1 4ZHX), with an r.m.s deviation of 0.520 over 869 Cα atoms (molecule 1 5EZV). The C2 molecules occupy the same binding space and γ-subunit residue interactions as the AMP/C2-bound structures (4ZHX). Importantly the structure reveals density for seven α1 RIM 2 residues (Fig. 5), including the main-chain of the nucleotide sensing α-E364 (α2 E368), further supporting our proposal that the α1 RIM2 interacts more strongly with the γ-subunit. Interestingly there was weak density for the carboxyl group of the E364 side-chain, possibly indicating a transient interaction with the γ-subunit residue R70.

## Discussion

In this study we showed that two topographically disparate drug sites on AMPK, one located at the classic α-kinase domain/β-CBM interface (ADaM site) and the other located within the solvent-accessible core of the γ-subunit (γ-pSite-1/γ-pSite-4), can be exploited to achieve synergistic activation of unphosphorylated α1-AMPK independent of AMP and upstream phosphorylation events. This provides a major advance to previous demonstrations of AMPK synergistic activation that have been dependent on AMP. Importantly, identification of C2-binding sites on the γ-subunit represents an entirely unexplored dimension for future rational drug design.

## Methods

### Reagents

Antibodies for pan AMPK α (#2793), FLAG (#2368) and HA (#2367); phosphospecific antibodies for α-pThr172 (#2535) and β-pSer108 (#4181), and λ-phosphatase (#P0753) were from Cell Signaling Technology. β1 antibody (#ab58175) and A769662 (#ab120335) were from Abcam. Anti-rabbit and anti-mouse IgG secondary antibodies fluorescently labelled with IR680 or IR800 dye, respectively, were from LI-COR Biosciences. Glutathione Sepharose 4B was from GE Life Sciences. FLAG peptide (DYKDDDK) was provided by GL Biochem (Shanghai), purified by reversed-phase chromatography and stored as lyophilized powder. All other reagents were purchased from Sigma.

### Constructs for crystallization

All mutants were generated using QuikChange Site-Directed Mutagenesis Kits (Stratagene), and constructs were sequence verified. *Escherichia coli*-expressed AMPK preparations were mass verified by time-of-flight (TOF) mass spectrometry. Heterotrimeric human AMPK His6-α2β1γ1 was expressed in *E. coli* strain Rosetta (DE3) using the pETDuet^TM^-1 expression system (Novagen) as previously described^13^. Briefly, cDNAs for α2 and γ1were sequentially inserted into pETDuet^TM^-1 multiple cloning sites (MCS) 1 (BamH1/Not1) and 2 (MfeI/XhoI), respectively, resulting in incorporation of an N-terminal hexahistidine tag onto α2. cDNA for β1 was inserted into pRSFDuet^TM^-1 MCS1 (NcoI/ BamHI). Heterotrimeric human AMPK α2/α1 RIM chimaera (α2(1-347)/α1(349-401)/α2(397-end) β1γ1)^16^ was expressed in *E. coli* strain Rosetta (DE3) using the pETDuet^TM^-1 and pCOLADuet^TM^ (Novagen). cDNAs for α2/1 chimaera and γ1were sequentially inserted into pETDUET^TM^-1 multiple cloning sites (MCS) 1 (BamHI/Not1) and 2 (MfeI/XhoI), respectively, resulting in incorporation of an N-terminal hexahistidine tag onto α2/1 chimaera. cDNA for β1 was inserted into pCOLADuet^TM^-1 MCS1 (NcoI/ BamHI).

### Protein expression and purification for crystallization

Expression cultures were grown in Luria-Bertani broth and induced at 16 °C with 0.25 mM isopropyl β-D-1-thiogalactopyranoside, before overnight incubation. Cells were lysed using a precooled EmulsiFlex-C5 homogenizer (Avestin) and AMPK purified using Nickel Sepharose and size exclusion chromatography in 50 mM Tris–HCl, pH 8.0, 150 mM NaCl, 2 mM Tris(2-carboxyethyl)phosphine buffer. AMPK was phosphorylated by incubation with CaMKK2 (expressed in Sf21 cells as a C-terminal FLAG fusion and purified on FLAG monoclonal antibody-coupled affinity resin^2^) in the presence of 2.5 mM MgCl_2_, 0.5 mM ATP and 0.5 mM AMP (1 h, 22 °C). Protein was re-purified using size exclusion chromatography, concentrated to ∼10 mg ml^−1^ and flash frozen in liquid nitrogen before storage at −80 °C (storage buffer: 50 mM Tris–HCl, pH 8.0, 150 mM NaCl, 2 mM Tris(2-carboxyethyl)phosphine).

### Crystallization

Full-length myristoylated and phosphorylated α2β1γ1 (4 mg ml^−1^) was mixed with a twofold molar excess of C2, AMP and S108tide (synthetic peptide: β1(102-117) R^115-117^, NH_2_-KLPLTRSHNNFVARRR-COOH) and a 1:1 molar ratio of Staurosporine and A769662, while full-length phosphorylated RIM swap chimaera (α2 1-347/α1 349-401/α2 397-end β1γ1)^16^ (4 mg ml^−1^) was mixed with a sixfold molar excess of C2, twofold molar excess of S108tide and a 1:1 molar ratio of Staurosporine and A769662. Crystals from both protein preparations were grown following the previously described method^8^. Briefly, protein was mixed equally at room temperature with a reservoir solution containing 8% PEG 3350, 0.1 M MgCl_2_, 1.0% glucose, 0.001% cocamidopropyl betaine and 0.1 M imidazole (pH 6.2). Protein crystals were incubated with reservoir solution containing an additional 5% glycerol, 5% PEG 400, 5% MPD, 5% sucrose, 5% sorbitol and 5% ethylene glycol for∼2 min before flash-cooling in liquid nitrogen. Data were collected at the Australian Synchrotron MX2 beamline, Melbourne, Australia. Data were processed and integrated using XDS^23^ and scaled using AIMLESS from the CCP4 suite^24^. The structure was solved by molecular replacement using Phaser from the CCP4 suite^25^ and 4CFE^8^ as the search model. Iterative rounds of model building and refinement were performed using Coot^26^ and Buster (https://www.globalphasing.com/buster/)^27^ respectively. C2 molecular coordinates and restraints were generated using the PRODRG web-server^28^. Omit maps were calculated using Sfcheck in the CCP4 suite^29^. Structure validation and protein–C2 interactions was performed using Molprobity^30^, figures were created using Pymol (https://www.pymol.org/). Data collection and refinement statistics are listed in Table 2.

### Protein expression and purification for AMPK enzyme assays

All mutants were generated using QuikChange Site-Directed Mutagenesis Kits (Stratagene), and constructs were sequence verified. Heterotrimeric AMPK was expressed in COS7 mammalian cells by transient transfection, using FuGENE HD (Promega) transfection reagent according to manufacturer's protocol. Transfected constructs included N-terminal GST-α1 and α2 fusions^2^ (pDEST27 expression vector), C-terminal FLAG- α1 and α2 fusions (cloned into pcDNA3.1 expression vector using XhoI/EcoRI (α1) or XhoI/HindIII (α2) restriction sites, C-terminal myc-β1 and β2 fusions^31^ (pcDNA3.1 expression vector) and N-terminal HA-γ1, γ2 and γ3 fusions^32^ (pMT2 expression vector). To assess C2 γ isoform selectivity and C2 kinetics (Fig. 1): GST-α1β1/2γ1/2/3 AMPK complexes were isolated from transfected lysates by immobilization on Glutathione Sepharose 4B (GE Life Sciences) prior to SAMS activity assay^2^. To assess C2/A769662 synergy (Fig. 2): GST-α1β1γ1 and GST-α2β1γ1 AMPK complexes were immobilized on Glutathione Sepharose 4B and incubated with either λ-phosphatase (50 mM Tris–HCl pH 7.4, 150 mM NaCl, 10% glycerol, 0.01% Tween-20 (buffer A) supplemented with 2 mM MnCl_2_) or CaMKK2 (buffer A supplemented with 2 mM MgCl_2_, 200 μM ATP) (1 h, 22 °C) prior to extensive washes with buffer A and elution in buffer A supplemented with 20 mM glutathione. The levels of α-pThr172 and β-pSer108 of treated AMPK complexes were assessed by immunoblot as described previously^32^ (Supplementary Fig. 2a). Briefly, membranes were simultaneously probed overnight at 4 °C with a phosphospecific primary antibody for α-pThr172 (1:2,000 dilution in phosphate buffered saline with 0.1% Tween-20 (v/v) (PBS-T)) or β-pSer108 (1:2,000 dilution in PBS-T) labelled with the fluorescent dye IR680 (LI-COR Biosciences) and probed for 1 h at room temp with pan α primary antibodies (1:1,000 dilution in PBS-T) or β1 primary antibodies (1:1,000 dilution in PBS-T) fluorescently labelled with IR800. Immunoreactive bands were visualized on an Odyssey membrane imaging system (LI-COR Biosciences) and densitometry analyses performed using integrated software. To evaluate the impact of γ-mutation on C2/AMP activity (Fig. 4): FLAG-α1(T172D)β1γ1 (wild-type and indicated mutants) AMPK complexes were immobilized on anti-FLAG M2 affinity Agarose gel (Supplementary Fig. 2b) before SAMS activity assay. The expression levels of the γ-mutants were assessed by immunoblot. Membrane was probed overnight at 4 °C with a FLAG primary antibody (1:2,000 dilution in PBS-T) labelled with the fluorescent dye IR680 (LI-COR Biosciences) and probed for 1 h at room temp with HA primary antibodies (1:1,000 dilution in PBS-T) fluorescently labelled with IR800 and analysed as above.

### AMPK activity assay

AMPK activity assay was conducted as described previously^13^. Briefly, AMPK complexes were purified as above and washed three times with a 40:1 v/v ratio of assay buffer/resin (assay buffer: 50 mM Hepes pH 7.4, 1 mM DTT and 0.02% Brij-35). Reaction mixtures (25 μl) containing 100 μM SAMS synthetic peptide (sequence: NH_2_-HMRSAMSGLHLVKRR-COOH), 5 mM MgCl_2_, 200 μM [γ-^32^P]-ATP were incubated AMPK for 10 min at 30 °C in the absence or presence of various C2 concentrations. AMP (100 μM) and/or A769662 (20 μM) were included as appropriate positive controls. After 10 min, phosphotransferase activity was quenched by spotting onto P81 ion-exchange chromatography paper (Whatman, GE Healthcare, Parramatta, NSW) followed by repeated washes in 1% H_3_PO_4_ (ref. 33). The level of ^32^P-transfer to the SAMS peptide was determined by liquid scintillation counting (Perkin Elmer, Melbourne, VIC). Statistical tests were conducted using analysis of variance (ANOVA). The values for EC_50_ and fold stimulation were calculated based on the equation: Activity=Basal+(((Fold stimulation x Basal)−Basal × [C2])/((EC_50_)+[C2])). Curve fitting was performed using KaleidaGraph software.

## Additional information

**Accession codes:** Coordinates and structure factors have been deposited in the protein data bank with the accession codes 4ZHX and 5EZV.

**How to cite this article:** Langendorf, C. G. *et al.* Structural basis of allosteric and synergistic activation of AMPK by furan-2-phosphonic derivative C2 binding. *Nat. Commun.* 7:10912 doi: 10.1038/ncomms10912 (2016).

## Supplementary Material

Supplementary Information Supplementary Figures 1-4, Supplementary Table 1 and Supplementary Notes 1-3.

## Figures and Tables

**Table 1:** Table summarizing basal activity of AMPK α1 and α2 and their fold activation to control, calculated from data in Fig. 2.

**Kinase**	**Treatment**	**Basal activity (nmol min**^**−1**^ **mg**^**−1**^**) (±s.e.m.)**	**Fold activation to control (**±**s.e.m.)**				
			**AMP**	**C2**	**A769662**	**AMP/A769662**	**C2/A769662**
α1	λ-Phosphatase	0.6±0.2	7.7±0.3	8.8±0.3	91.8±5.9	1100±77	1040±43
	CaMKK2	699.8±26.1	1.7±0.1	2.5±0.1	1.5±0.1	1.0±0.1	1.1±0.1
α2	λ-Phosphatase	1.2±0.4	3.9±0.5	2.1±0.3	1.1±0.1	6.1±0.1	1.2±0.2
	CaMKK2	697.6±36.6	1.8±0.1	1.9±0.1	7.7±0.3	5.4±0.5	6.8±0.3

**Table 2:** Data collection and refinement statistics.

*Data collection*	4ZHX	5EZV
Space group	*P*2_1_	*P*2_1_
Cell dimensions		
a, b, c (Å)	76 134.2 141.5	75.6 134.1 141.3
α, β, γ (°)	90 93.0 90	90 93.2 90
Resolution (Å)	48.66–2.99 (3.07-2.99)*	49.3–2.99 (3.08-2.99)
*R*_pim_ (%)	0.071 (0.48)	0.053 (0.31)
*R*_merge_ (%)	0.096 (0.61)	0.081 (0.47)
<*I/σ*(*I*)>	8.1 (2.2)	10.1 (2.6)
CC_1/2_	0.98 (0.56)	0.99 (0.77)
Completeness	98.8 (99.4)	99.4 (98)
Redundancy	3.2 (3.2)	3.2 (3.2)
Wilson B	83.8	87.4
		
*Refinement statistics*		
Resolution (Å)	43.78–2.99	49.3–2.99
*No. of reflections*		
Total	183,106	182,062
Unique	56,598	56,584
*R*_work_*/R*_free_	22.6/24.4	22.4/24.5
*No. of atoms*		
Protein	14,501	14,403
Ligand/ion	382	328
Water	142	104
*B-factors*		
Overall	69.7	66.5
Protein	70	67.6
Ligand	56.2	62.8
Water	42.3	51.9
*R.m.s. deviations*		
Bond lengths (Å)	0.007	0.008
Bond angles (°)	0.94	0.98
*Molprobity*		
Clash score	2.55 (100th percentile)	5.74 (100th percentile)
*Ramachandran*		
Favoured	96.30%	97.37%
Outliers	0.28%	0.05%
Protein geometry	1.38 (100th percentile)	1.43 (100th percentile)

^*^Highest resolution shell is shown in parenthesis. Each data set was obtained from a single crystal.
