# Supplementary material for: Structural basis of allosteric and synergistic activation of AMPK by furan-2-phosphonic derivative C2 binding
Source: Nat Commun. 2016 Mar 8;7:10912. doi: 10.1038/ncomms10912 (PMC4786773; doi:10.1038/ncomms10912)

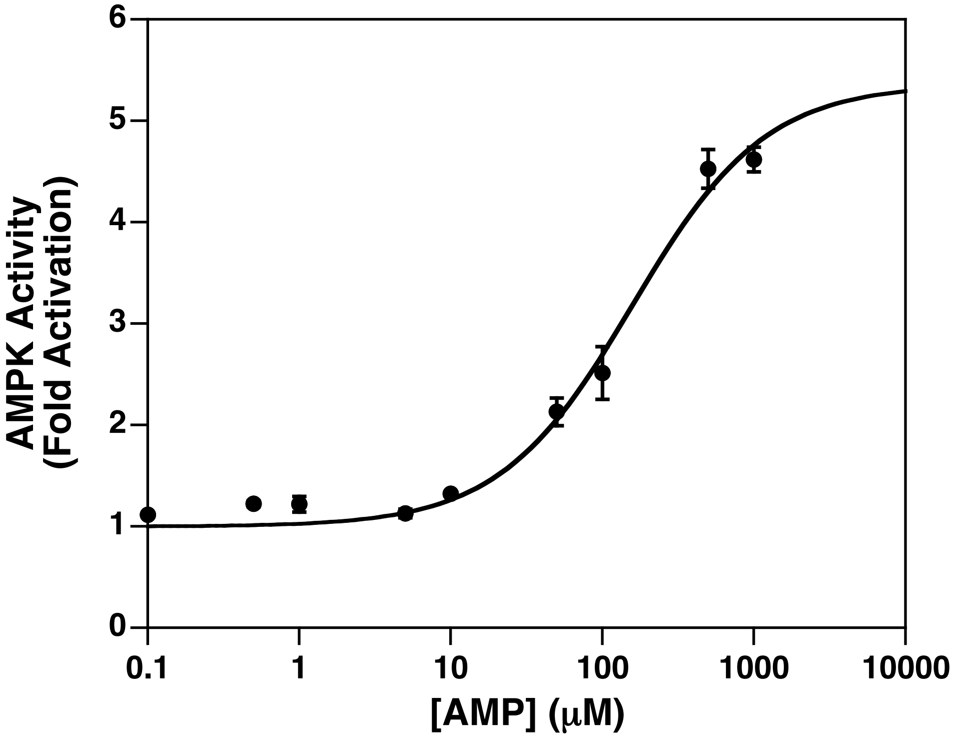

Supplementary Figure 1: Effect of high ATP concentration on AMP activation of AMPK α1. AMP dose-response (0 – 1 mM) at near-physiological (2 mM) ATP concentration; results from six independent experiments were plotted as a dose-response curve of AMPK activity (fold activation ± SEM) vs [AMP] (μM). The values for EC_{50}, fold stimulation and Hill coefficients were calculated based on fitting the data to the equation: Activity = Basal + (((Fold stimulation x Basal) – Basal x $[C2]^h$)/ $((EC_{50})^h$ + $[C2]^h$)).

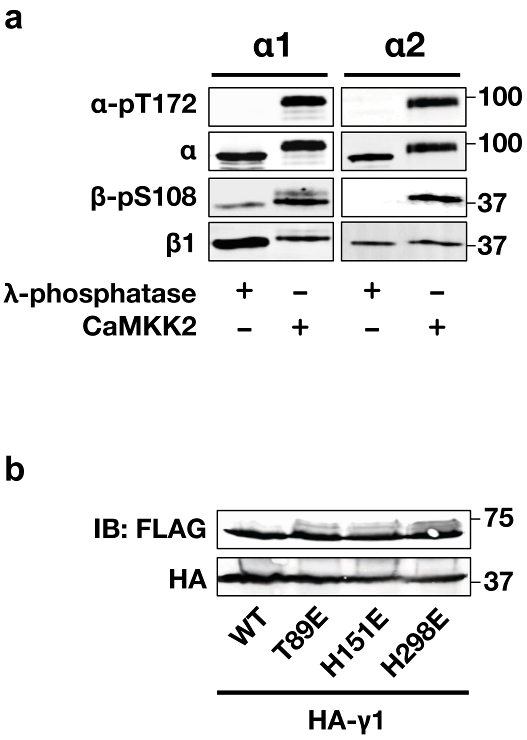

Supplementary Figure 2: Western blotting of AMPK protein. (a) Western blot of AMPK α1 and α2 showing the levels of AMPK α-pT172, total α-AMPK, β-pS108 and total β1-AMPK following treatment with λ-phosphatase or CaMKK2. **(b)** Expression of AMPK γ1 WT and its three respective mutants (γT89E, γH151E and γH298E). Levels were detected using immunoblotting with FLAG-antibody for AMPK α-subunit and HA-tag antibody for γ-subunit. Images have been cropped to improve clarity of the figure. Original blots were displayed in Appendix Ai-iii.

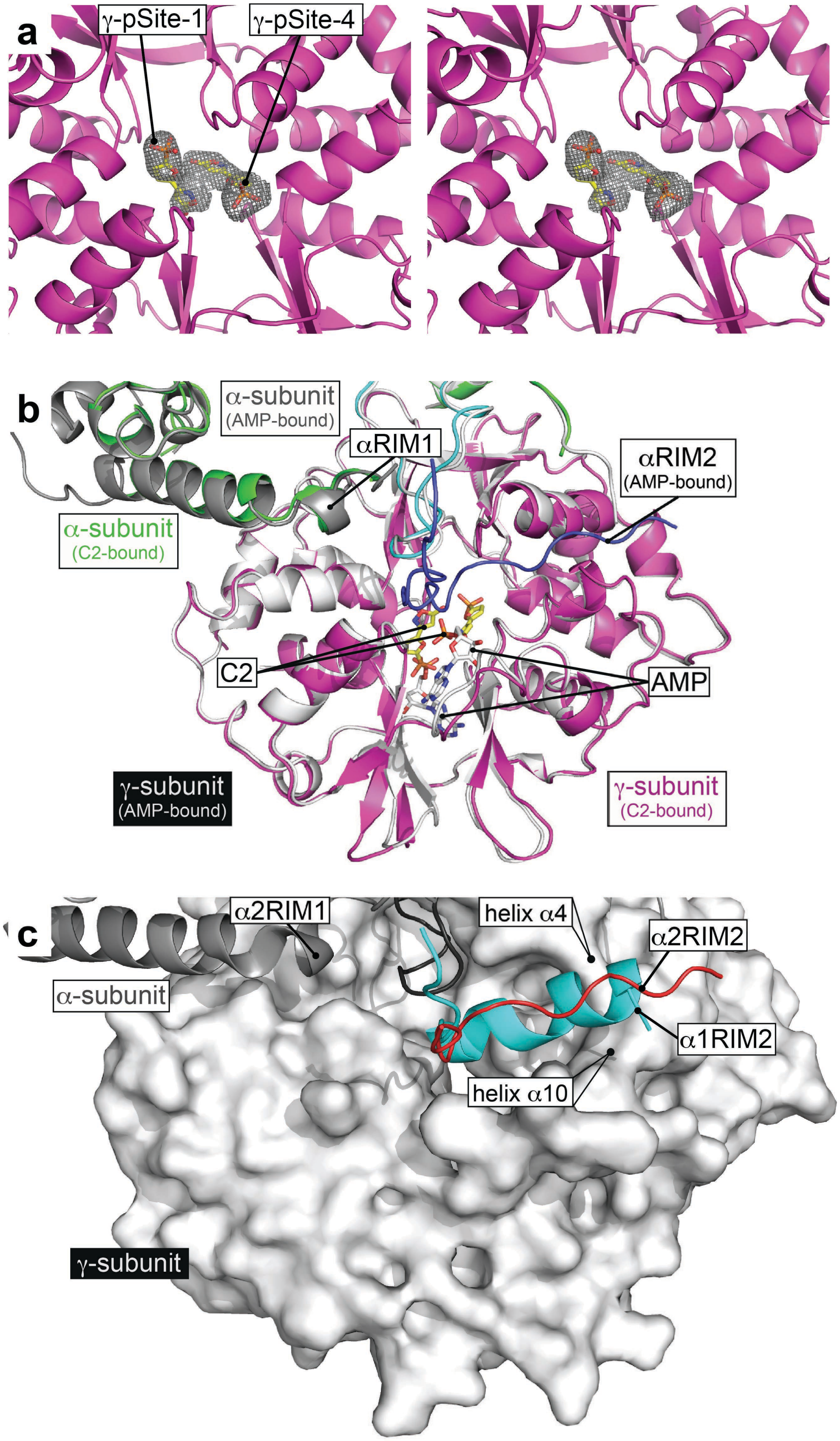
a
γ-pSite-1
γ-pSite-4
b
α-subunit
(AMP-bound)
αRIM1
αRIM2
(AMP-bound)
α-subunit
(C2-bound)
C2
AMP
γ-subunit
(AMP-bound)
γ-subunit
(C2-bound)
c
α2RIM1
helix α4
α2RIM2
α-subunit
α1RIM2
helix α10
γ-subunit

Supplementary Figure 3: αRIM interactions with the γ-subunit. (**a**) Stereo omit electron density map ($2F_o$-F_c) for the C2 molecules, contoured at 2.0 σ (gray mesh). (**b**) Differences between C2-bound (α = green; γ = magenta) and AMP-bound (α = dark gray; γ = white) heterotrimers. Electron density for the αRIM2 was only visible in the AMP-bound heterotrimer. (**c**) Comparison of the αRIM2 motif of AMPK isoforms; superposition of the αRIM2 motif from α1 (cyan; PDB 4RER) and α2 (red; PDB 4CFE), with γ1 from a α2β1γ1 (PDB 4CFE) shown in surface representation.

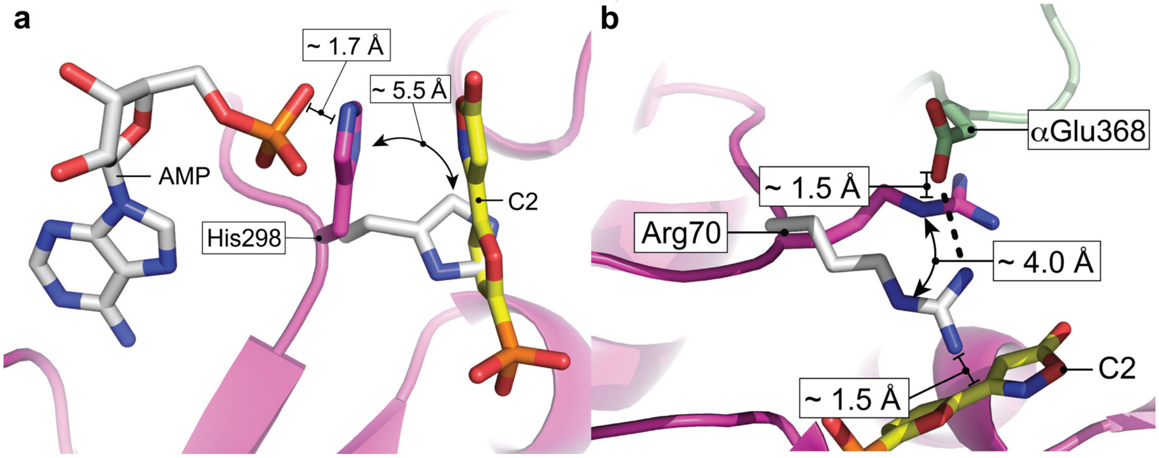

Supplementary Figure 4: C2 and AMP binding to the γ-subunit causes distinct conformational changes. The γ-subunit (magenta) is shown in cartoon representation, and C2 (yellow), AMP (grey) and selected side-chains (α = green; γ = grey) in stick representation. (**a**) Two conformations of γHis298. The conformational differences are necessary to avoid a clash with AMP (white γHis298) or C2 (magenta γHis298). (**b**) Two conformations of γArg70. Binding of C2 disrupts the hydrogen bond (black dashed line) between γArg70 and αGlu368 (AMP-bound; white and green, respectively). The close contact with C2 moves γArg70 (magenta) ~ 4.0 Å, clashing with αGlu368 (C2-bound; not modeled) in this new conformation and disengaging the αRIM.

Supplementary Table 1: Values for EC_{50}, Vmax and Hill coefficients for C2 **(Fig. 1d)** and AMP **(Supplementary Fig. 1)** at near-physiological ATP (2 mM), calculated based on fitting the data to the equation: Activity = Basal + (((Fold stimulation x Basal) – Basal x $[C2]^h$)/ $((EC_{50})^h + [C2]^h)$).

ATP (2mM)	C2	AMP
EC_{50} (unit)	50.3 nM	158.1 μM
Fold stimulation	6.7 ± 0.2	5.4 ± 0.6
Hill coefficient	2.3 ± 0.4	1.0 ± 0.2

Supplementary Note 1: Western blot of AMPK α1 showing the levels of AMPK α-pT172, total α-AMPK, β-pS108 and total β1-AMPK following treatment with λ-phosphatase or CaMKK2 **(Supplementary Fig. 2a)**. All blots displayed are uncropped scans from Odyssey phosphoimager.

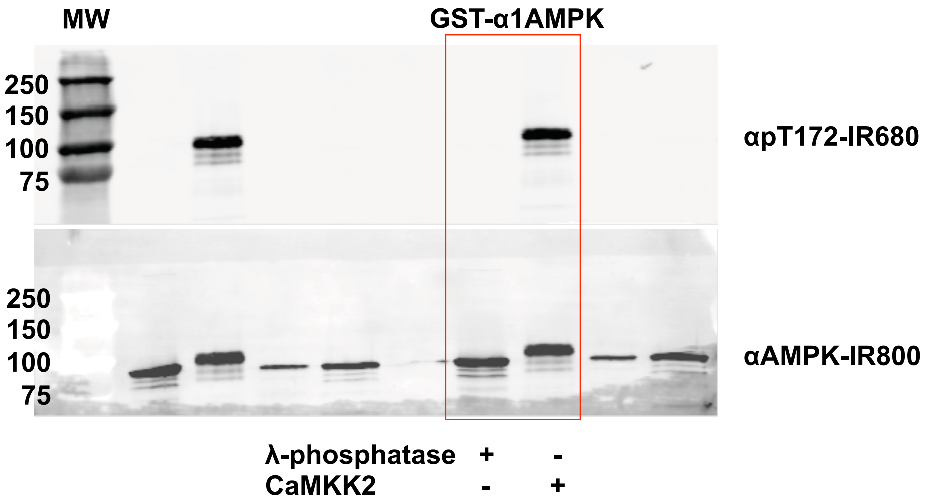

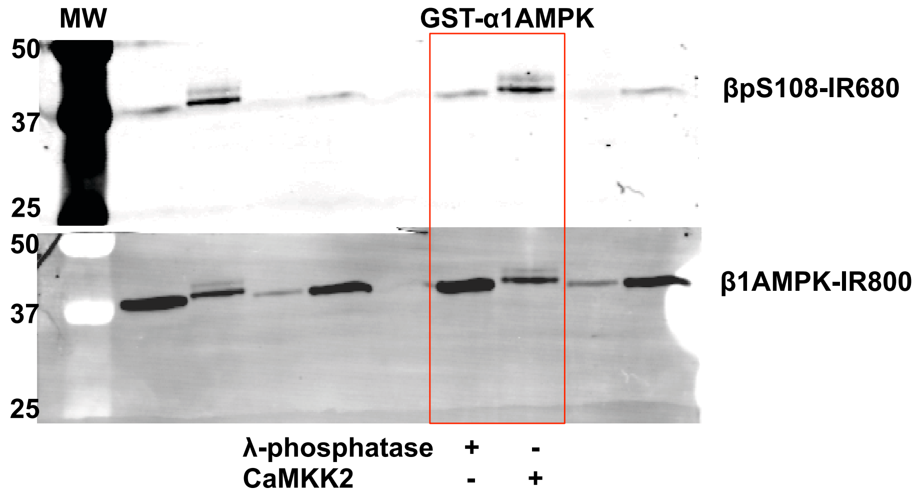

Supplementary Note 2: Western blot of AMPK α2 showing the levels of AMPK α-pT172, total α-AMPK, β-pS108 and total β1-AMPK following treatment with λ-phosphatase or CaMKK2 **(Supplementary Fig. 2a)**. All blots displayed are uncropped scans from Odyssey phosphoimager.

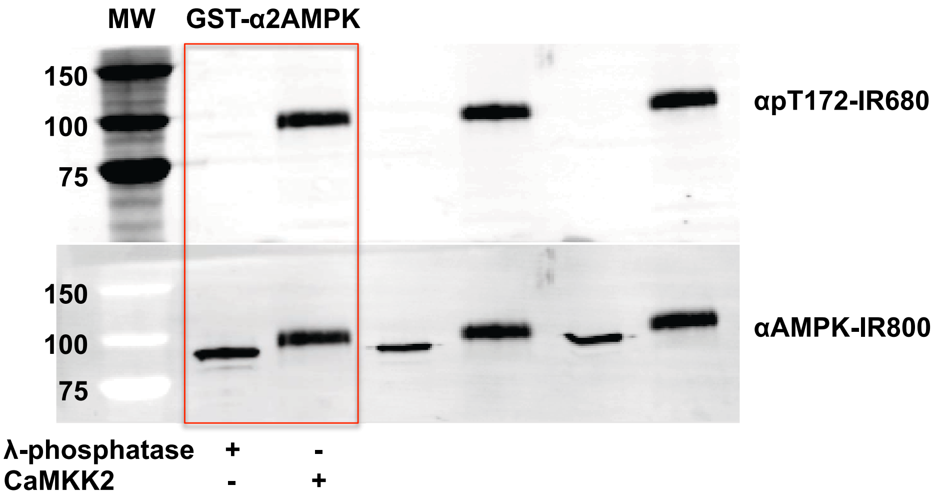

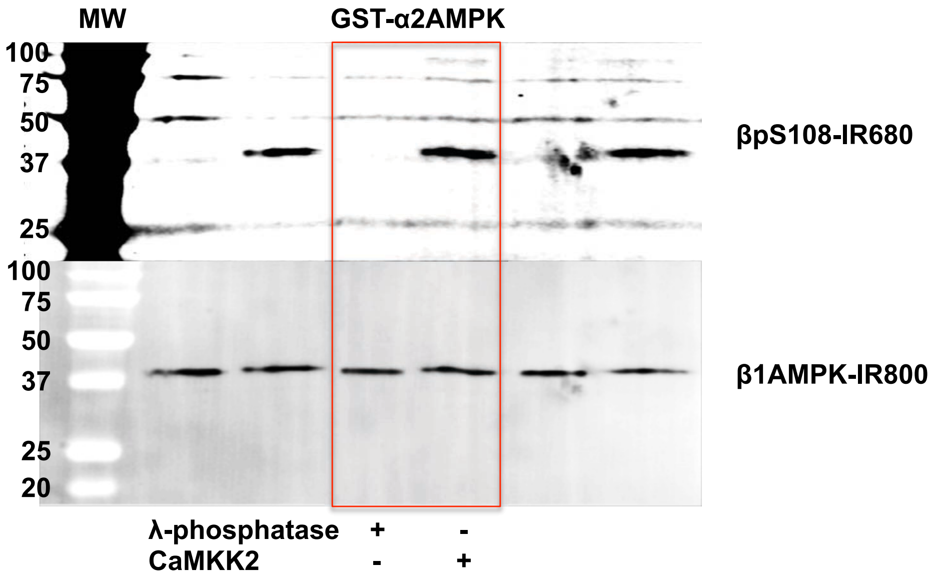

Supplementary Note 3: Western blot of the expression of AMPK γ1 WT and its three respective mutants (γT89E, γH151E and γH298E; **Supplementary Fig. 2b**), with appropriate control (GSTα-AMPK) included. Levels were detected using immunoblotting with FLAG-antibody for AMPK α-subunit and HA-tag antibody for γ-subunit. All blots displayed are uncropped scans from the Odyssey phosphoimager.

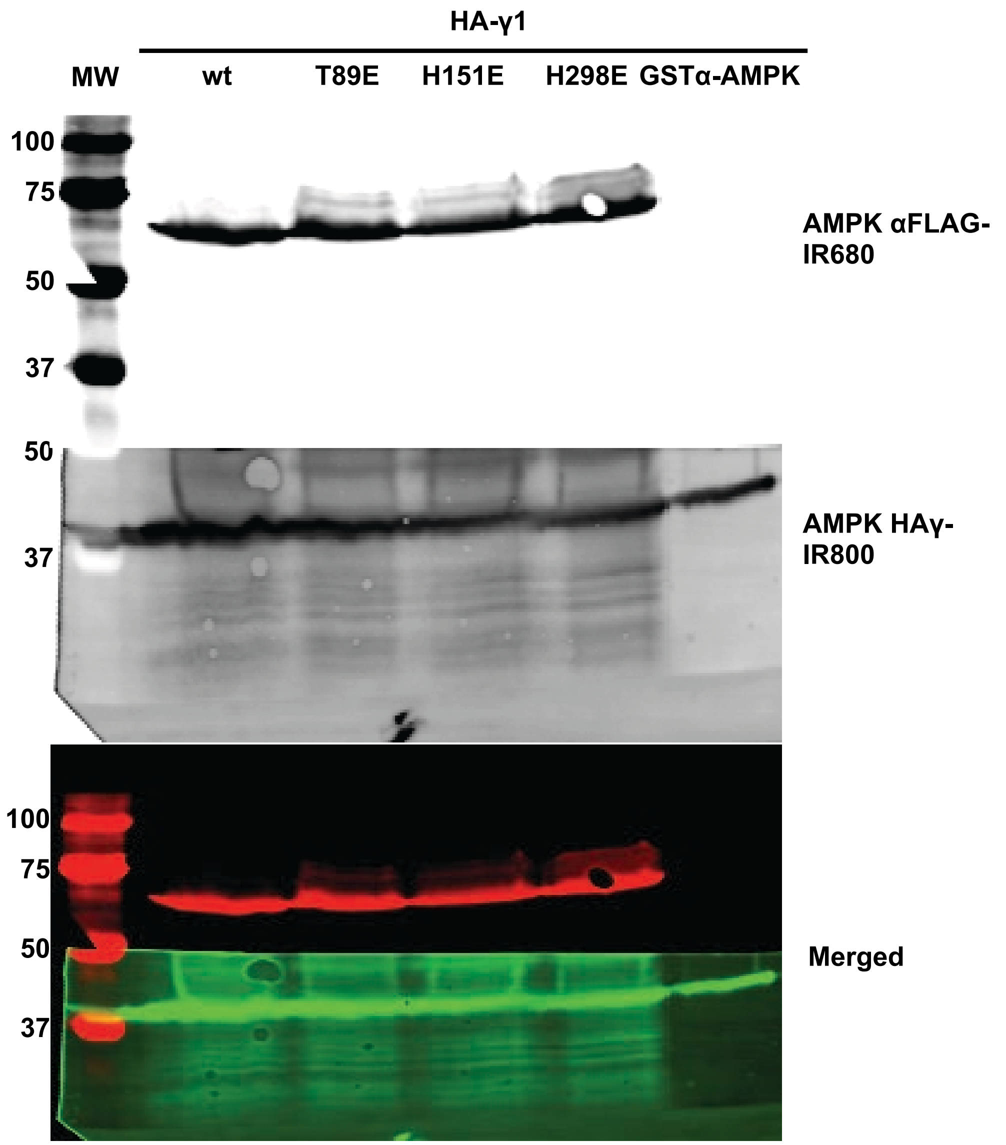

Supplement: Supplementary Information — Supplementary Figures 1-4, Supplementary Table 1 and Supplementary Notes 1-3. [file ncomms10912-s1.pdf]
